# Differential regulations of fibronectin and laminin in Smad2 activation in vascular endothelial cells in response to disturbed flow

**DOI:** 10.1186/s12929-017-0402-4

**Published:** 2018-01-02

**Authors:** Tung-Lin Yang, Pei-Ling Lee, Ding-Yu Lee, Wei-Li Wang, Shu-Yi Wei, Chih-I Lee, Jeng-Jiann Chiu

**Affiliations:** 1 Department of Life Sciences, National Central University, Jung-Li, Taoyuan, Taiwan; 2 Institute of Cellular and System Medicine, National Health Research Institutes, Miaoli, Taiwan; 3 Departments of Food Science and Biological Science and Technology, China University of Science and Technology, Taipei, Taiwan; 4 Institute of Biomedical Engineering, National Tsing Hua University, Hsinchu, Taiwan; 5 Institute of Biomedical Engineering, National Cheng-Kung University, Tainan, Taiwan; 6 College of Pharmacy, Taipei Medical University, Taipei, Taiwan

**Keywords:** Endothelial cell, Extracellular matrix, Integrin, Shear stress, Transforming growth factor receptor

## Abstract

**Background:**

Atherosclerosis occurs in arterial curvatures and branches, where the flow is disturbed with low and oscillatory shear stress (OSS). The remodeling and alterations of extracellular matrices (ECMs) and their composition is the critical step in atherogenesis. In this study, we investigated the effects of different ECM proteins on the regulation of mechanotransduction in vascular endothelial cells (ECs) in response to OSS.

**Methods:**

Through the experiments ranging from in vitro cell culture studies on effects of OSS on molecular signaling to in vivo examinations on clinical specimens from patients with coronary artery disease (CAD), we elucidated the roles of integrins and different ECMs, i.e., fibronectin (FN) and laminin (LM), in transforming growth factor (TGF)-β receptor (TβR)-mediated Smad2 activation and nuclear factor-κB (NF-κB) signaling in ECs in response to OSS and hence atherogenesis.

**Results:**

OSS at 0.5±12 dynes/cm^2^ induces sustained increases in the association of types I and II TβRs with β1 and β3 integrins in ECs grown on FN, but it only transient increases in ECs grown on LM. OSS induces a sustained activation of Smad2 in ECs on FN, but only a transient activation of Smad2 in ECs on LM. OSS-activation of Smad2 in ECs on FN regulates downstream NF-κB signaling and pro-inflammatory gene expression through the activation of β1 integrin and its association with TβRs. In contrast, OSS induces transient activations of β1 and β3 integrins in ECs on LM, which associate with type I TβR to regulate Smad2 phosphorylation, resulting in transient induction of NF-κB and pro-inflammatory gene expression. In vivo investigations on diseased human coronary arteries from CAD patients revealed that Smad2 is highly activated in ECs of atherosclerotic lesions, which is accompanied by the concomitant increase of FN rather than LM in the EC layer and neointimal region of atherosclerotic lesions.

**Conclusions:**

Our findings provide new insights into the mechanisms of how OSS regulates Smad2 signaling and pro-inflammatory genes through the complex signaling networks of integrins, TβRs, and ECMs, thus illustrating the molecular basis of regional pro-inflammatory activation within disturbed flow regions in the arterial tree.

## Background

Atherosclerotic lesions are prone to localize at arterial branches and curvatures, which are constantly exposed to disturbed flow with low and oscillatory shear stress (OSS). Therefore, OSS has a significant impact on the vascular endothelial cells (ECs) of these regions [1]. OSS not only can increase the expression of pro-inflammatory molecules in ECs, but also enhances the adhesion of circulating monocytes to ECs. These OSS-induced EC dysfunctions are the initiation steps leading to vascular pathologies, including atherosclerosis [1]. Several reports indicate that extracellular matrix (ECM) remodeling and ECM composition change in the arterial wall are key processes that can induce an activated EC phenotype, stimulate the proliferation of smooth muscle cells, and promote pro-inflammatory signaling within the development of atherosclerosis [1–3]. In normal conditions, ECs adhere to the vascular basement membrane, whose major components of ECM are laminin (LM), collagen (Coll.) IV, and entactin/nidogen. When atherosclerosis develops, ECs are subjected to injury, inflammation, and angiogenesis, which cause vascular remodeling leading to ECM deposition of fibronectin (FN) and fibrinogen into the subendothelial matrix [1–3]. Integrins are the main receptors to interact with ECM proteins [1, 4, 5]. They are transmembrane proteins that compose of α and β chains to form heterodimer complexes. For example, integrins α5β1 and ανβ3 are the major FN receptors on ECs, whereas integrin α6β1 is the major LM receptor on ECs [1, 6]. Recent studies indicate that integrins are mechanosensitive receptors that can modulate cellular signaling and functions [1, 7]. When ECs are exposed to OSS, the ECM-integrin signaling is constitutively activated. This ECM-integrin signaling includes the activations of focal adhesion kinase (FAK), Shc, mitogen-activated protein kinases (MAPKs), and Rho family GTPases [1, 2, 7]. OSS-induced ECM-integrin signaling results in the activation of nuclear factor-κB (NF-κB), which is an atherogenic transcription factor involved in regulating pro-inflammatory genes in ECs [2, 8].

The signaling of TGF-β receptors (TβRs) also plays important roles in the development of atherosclerosis [9, 10]. The two main TGF-β receptors, type І (TβRІ) and type ІІ (TβRІІ), have been identified to form a heteromeric complex to regulate the phosphorylation of Smads (i.e., Smad2/3), which subsequently regulate transcriptions of several genes involved in cell proliferation, migration, and atherogenesis [10–12]. However, it is not clear whether TβRs can serve as mechanoreceptors in the collaboration of integrins to transmit mechanical stimulation into intracellular Smads signaling in ECs grown on different ECMs, including LM (normal condition) and FN (disease condition) [13, 14]. Our previous studies have demonstrated that bone morphogenetic protein receptor (BMPR) type II (BMPRII) can modulate the OSS-induced Smad1/5 activation and its downstream signaling through the modulation of BMPR type IB (BMPRIB)-ανβ3 integrin association [15, 16]. This finding suggested that BMPRs are important mechanoreceptors that can interact with integrins to modulate cell signaling. Here we postulated that TβRs may also serve as important mechanoreceptors like BMPRs to regulate mechanotransduction in ECs, and that OSS may activate TβR-specific Smad2 to induce EC inflammation through the interactions between TβRs and integrins.

In the present study ranging from in vitro cell culture studies on effects of disturbed flow on molecular signaling to in vivo investigations on clinical specimens from patients with coronary artery disease (CAD), we investigated the roles of integrins and different ECMs, including FN and LM, in TGFβ receptor-mediated Smad2 activation and NF-κB signaling in ECs in response to disturbed flow. We found that FN and LM play differential roles in modulating OSS-induced Smad2 activation and pro-inflammatory gene expression in ECs. These differential effects of FN and LM on EC Smad2 signaling and pro-inflammatory responses may be attributable to the differential regulations in the activation of various integrins and their association with TβRs. In vivo investigations on the clinical specimens from CAD patients demonstrated that Smad2 is highly activated in ECs of atherosclerotic lesions, which is accompanied by the concomitant increase of FN rather than LM expression in lesions. Our findings provide new insights into the mechanisms by which TβR-specific Smad2 serves as a connecting link for the chain of events of disturbed flow, mechanical sensing, EC inflammation, and atherogenic responses in the arterial wall. Such findings may provide insights to therapeutic intervention against disturbed flow-associated vascular disorders, e.g., atherosclerosis.

## Methods

### Materials

Goat polyclonal antibody (pAb) against Smad2/3, mouse monoclonal antibodies (mAbs) against TGFβRII and β3 integrin, and rabbit pAb against TGFβRI were purchased from Santa Cruz Biotechnology. Rabbit pAb against phospho-Smad2 (Ser465/467) was purchased from Cell Signaling Technology. Mouse mAbs against human integrin β1 and HUTS4 were purchased from Chemicon. Mouse mAb against human LIBS2 was purchased from Millipore. The control siRNA and specific siRNAs of Smad2, β1 integrin, β3 integrin, TGFβRII, and TGFβRI were purchased from Invitrogen. FN was purchased from BD Biosciences. LM was purchased from Sigma. All other chemicals of reagent grade, unless noted, were obtained from Sigma.

### Cell cultures

ECs were isolated from fresh human umbilical cords and cultured in M199 (Gibco, Grand Island, NY) supplemented with 20% FBS (Gibco) and 1% penicillin/streptomycin (Gibco). These cells were grown in Petri dishes for 3 days, after which the cells were typsinized and then seeded onto glass slides (75 by 38 mm; Corning) pre-coated with different ECM proteins, including FN and LM (30 g/ml for each) to reach confluence (~ 1–2 × 10^5^ cells/cm^2^). The culture medium was then exchanged with a medium that was identical to the previous medium except that it contained only 2% FBS, and the cells were further incubated for 24 h prior to the experiment.

### Flow apparatus

The cultured ECs were subjected to shear stress in a parallel-plate flow chamber, as described previously [15, 16]. The flow channel in the chamber was created by a silicon gasket with dimensions of 2.5 cm for channel width (w) and 0.025 cm for channel height (h). The fluid shear stress (τ) generated on the cells seeded on the glass slide can be estimated as τ = 6Qμ/wh^2^, where Q is the flow rate and μ is the dynamic viscosity of the perfusate. The oscillatory flow comprises a low level of mean flow with shear stress at 0.5 dynes/cm^2^ supplied by a hydrostatic flow system to provide the basal nutrient and oxygen delivery, and the superimposition of a sinusoidal oscillation using a piston pump with a frequency of 1 Hz and peak-to-peak amplitude of ±4 dynes/cm^2^. The static control cells subjected to the same time protocol in terms of incubation media and other conditions as the experimental cells, except that they were not subjected to flow.

### Western blot analysis

Cells were lysed with a buffer containing 1% Nonidet P-40, 0.4% sodium deoxycholate, 0.1% SDS, and a protease inhibitor mixture (PMSF, aprotinin, and sodium orthovanadate). Detailed procedures of the experiments are previously described [15, 16].

### Immunoprecipitation

ECs were lysed with a buffer containing 25 mM Hepes (pH 7.4), 1% Triton X-100, 1% deoxycholate, 0.1% SDS, 0.125 M NaCl, 5 mM EDTA, 50 mM NaF, 1 mM Na3VO4, 1 mM PMSF, 10 mg/mL leupeptin, and 2 mM β-glycerophosphate (BGP). Detailed procedures of the experiments are previously described [15, 16].

### RNA isolation and RT-PCR

The total RNA was extracted via the TRIzol Reagent (Invitrogen) according to the manufacturer’s instructions and converted to cDNA. The primer sequences were designed as NF-κB (sense: 5´-TGGGATCTGCACTGTAACTG-3′; antisense: 5´-CAAATCCTTCCCAGACTCCA-3′; product length, 513 bp); intercellular adhesion molecule (ICAM)-1 (sense: 5´-ATTATGACTGCGGCTGCTACC-3′; antisense: 5´-CGACTGGACGACAGGAGTTGT-3′; product length, 290 bp); vascular cell adhesion molecule (VCAM)-1 (sense: 5´-CTACACTTTTGATTTCTGTG-3′; antisense: 5´-GGAAGTGGAATTAATTATCCAA-3′; product length, 450 bp); E-selectin (sense: 5´-GTGAACCCAACAATAGGCAA-3′; antisense: 5´-CAGGTGAAGTTGCAGGATGA-3′; product length, 686 bp); and GAPDH (sense: 5´-CCACCCATGGCAAATTCCATGGCA-3′; antisense: 5´-TCTAGACGGCAGGTCAGGTCCACC-3′; product length, 597 bp). Detailed procedures of the experiments are previously described [15, 16].

### siRNA and transfection

For siRNA transfection, ECs at 70–80% confluence were transfected with the designated siRNA at 30 nM for 48 h using the RNAiMAX transfection kit (Invitrogen). Detailed procedures of the experiments are previously described [15, 16].

### Human coronary arteries and immunohistochemistry

Six diseased human coronary arteries and five control internal thoracic arteries were harvested from five patients with end-stage heart failure undergoing heart transplantation at Tri-Service General Hospital in Taipei, as approved by the Hospital Human Subjects Review Committee (IRB No.: 1–103–05-132). Samples were fixed and embedded in paraffin blocks for immunohistochemical studies. Three serial sections were performed for phospho-Smad2, FN, and LM, respectively, and were counterstained with the EC marker von Willebrand factor (vWF) and the nuclear marker 4′,6-diamidino-2-phenylindole (DAPI). Briefly, serial sections (5 μm thick) of human coronary and internal thoracic arteries were placed on poly-L-lysine-coated slides, deparaffinized, and blocked for 1 h with PBS containing 5 mg/mL of serum albumin. One section was incubated with anti-phospho-Smad2 antibody (1:50) for 1 h at 37 °C, followed by rhodamine-conjugated secondary antibody (1:100) in the presence of DAPI for 2 h at room temperature. The second and third sections were incubated with an antibody (1:50 each) against laminin or fibronectin, respectively, followed by the secondary antibodies for 2 h at room temperature. Images were acquired and analyzed using a Zeiss fluorescence microscope with Axiovision image analysis software.

### Statistical analysis

Results are expressed as mean ± SEM. Statistical significance was determined by using Student’s t-test for two groups of data and one-way analysis of variance (ANOVA) followed by the Scheffe’s test for multiple comparisons. The level of statistical significance was defined as *P* < 0.05 from 3 or 4 separate experiments.

## Results

### Oscillatory flow induces a sustained phosphorylation of Smad2 in ECs grown on FN, but a transient phosphorylation of Smad2 in ECs grown on LM.

**Fig. 1:**
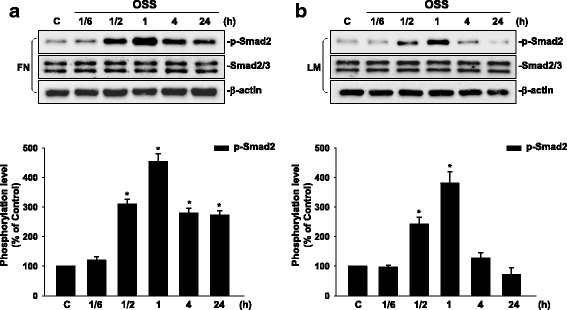
OSS induces a sustained phosphorylation of Smad2 in ECs plated on FN, whereas it only induces a transient phosphorylation of Smad2 in ECs plated on LM. ECs grown on FN (**a**) or LM (**b**) were kept under static conditions (C) or subjected to OSS (0.5 ± 4 dyn/cm^2^) for 1/6 h (10 min), 1/2 h (30 min), 1 h, 4 h, and 24 h. The phosphorylation of Smad2 was determined by using Western blot analysis. Data are means ± SEM from three independent experiments. ^*^P < 0.05 vs. static control cells

We first investigated whether different extracellular matrices, i.e., FN and LM, affect Smad2 activation in ECs in response to OSS. ECs were plated on FN or LM, and then subjected to OSS over a time course of 24 h. In the ECs plated on FN, the phosphorylation of Smad2 was induced to reach a maximal level within 1 h by OSS and remained elevated after 24 h of shearing in comparison to static control cells (Fig. 1a). In contrast, the phosphorylation of Smad2 induced by OSS in ECs plated on LM was transient and returned to the basal level after 24 h of shearing (Fig. 1b). These results showed that OSS induces a sustained activation of Smad2 in ECs plated on FN, but only a transient activation of Smad2 in ECs plated on LM, indicating that different ECM proteins play a differential role in the modulation of Smad2 mechanotransduction in vascular ECs in response to disturbed flow.

### Oscillatory flow induces sustained activations of β1 and β3 integrins in ECs grown on FN, but only transient activations of these integrins in ECs grown on LM.

**Fig. 2:**
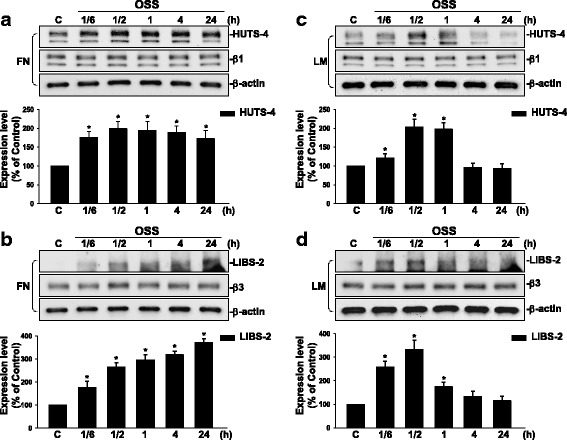
OSS induces sustained activations of β1 and β3 integrins in ECs plated on FN, but only transient activations of these integrins in ECs plated on LM. ECs plated on FN (**a** and **b**) or LM (**c** and **d**) were kept under static conditions (C) or subjected to OSS (0.5 ± 4 dyn/cm^2^) for 1/6 h (10 min), 1/2 h (30 min), 1 h, 4 h, and 24 h; Western blot analysis was used to determine the activations of the β1 and β3 integrins by using HUTS-4 and LIBS-2 antibodies, respectively. Data are means ± SEM from three independent experiments. ^*^P < 0.05 vs. static control cells

Integrins have been shown to be mechanoreceptors for regulating cell functions [1, 5, 8]. We investigated the effect of OSS on the activations of β1 and β3 integrins in ECs plated on different ECM proteins. ECs were cultured on FN or LM, and then exposed to OSS over a time course of 24 h. EC lysates were subjected to immunoblotting with anti-HUTS-4 and anti-LIBS-2 antibodies, which binds to the ligand-bound β1 and β3 integrins, respectively. The results showed that β1 (Fig. 2a) and β3 integrins (Fig. 2b) were activated within 1/6 h and reached the maximal level within 1/2 h in comparison to static control cells. These integrin activations were sustained for 24 h in ECs plated on FN after exposure to OSS. In contrast, OSS-activations of β1 (Fig. 2c) and β3 integrins (Fig. 2d) in ECs on LM were transient and returned to the basal level after 4 h of shearing. These differential activations of FN and LM on β1 and β3 integrins in ECs in response to OSS may contribute to their differential regulations in OSS-induced Smad2 activation in ECs.

### Oscillatory flow-induced Smad2 activation is regulated by β1 integrin in ECs grown on FN, and by β1 and β3 integrins in ECs grown on LM.

**Fig. 3:**
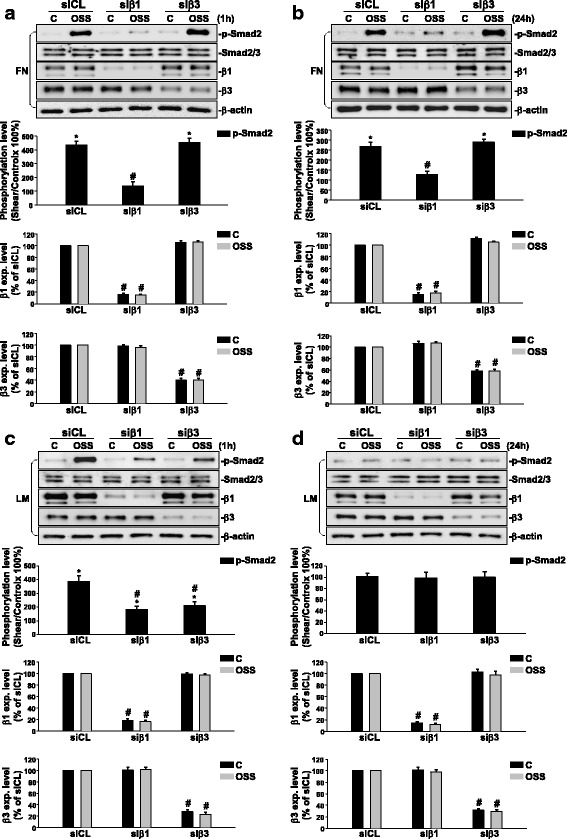
OSS-activation of Smad2 is regulated by β1 integrin in ECs plated on FN, and by β1 and β3 integrins in ECs plated on LM. ECs plated on FN (**a** and **b**) or LM (**c** and **d**) were transfected with control siRNA (siCL) or specific siRNA (30 nM each) of β1 (siβ1) or β3 (siβ3) integrin for 48 h. The cells were kept under static conditions (C) or subjected to OSS (0.5 ± 4 dyn/cm^2^) for 1 h (**a** and **c**) or 24 h (**b** and **d**). The phosphorylation of Smad2 and expressions of β1 and β3 integrins were determined by using Western blot analysis. Data are means ± SEM from three independent experiments. ^*^P < 0.05 vs. static control cells. ^#^*P* < 0.05 vs. siCL-transfected cells

To examine whether OSS-induced integrin activation could affect Smad2 phosphorylation in ECs plated on FN and LN, ECs were transfected with β1 or β3 integrin-specific siRNAs (30 nM), and kept under static conditions or exposed to OSS for 1 h or 24 h. We found that ECs transfected with β1 integrin-specific siRNA, which caused an approximately 80% reduction (compared with control siRNA) in β1 integrin protein expression, could significantly inhibit OSS-induced Smad2 phosphorylation in ECs on FN when shearing for 1 h (Fig. 3a) or 24 h (Fig. 3b). However, this OSS-induced EC Smad2 phosphorylation could not be inhibited by β3 integrin-specific siRNA, which caused an approximately 50–70% reduction in β3 integrin protein expression compared with the cells transfected with control siRNA (Fig. 3a and Fig. 3b). On the other hand, ECs transfected with both β1 or β3 integrin-specific siRNAs (compared with control siRNA) could result in significant inhibitions in OSS-induced Smad2 phosphorylation in ECs plated on LM when shearing for 1 h (Fig. 3c). Since OSS-induced Smad2 phosphorylation in ECs on LM returned to the basal level after 24 h of shearing (Fig. 1b), the transfection with either β1 or β3 integrin-specific siRNA had no effect on this OSS-mediated Smad2 responsiveness (Fig. 3d). Taken together, our results showed that OSS-induced Smad2 phosphorylation in ECs was regulated by both β1 and β3 integrins on LM, but was regulated by only β1, but not β3, integrin on FN.

### Oscillatory flow-induced Smad2 activation is modulated by TGFβ receptors in ECs plated on either FN or LM.

**Fig. 4:**
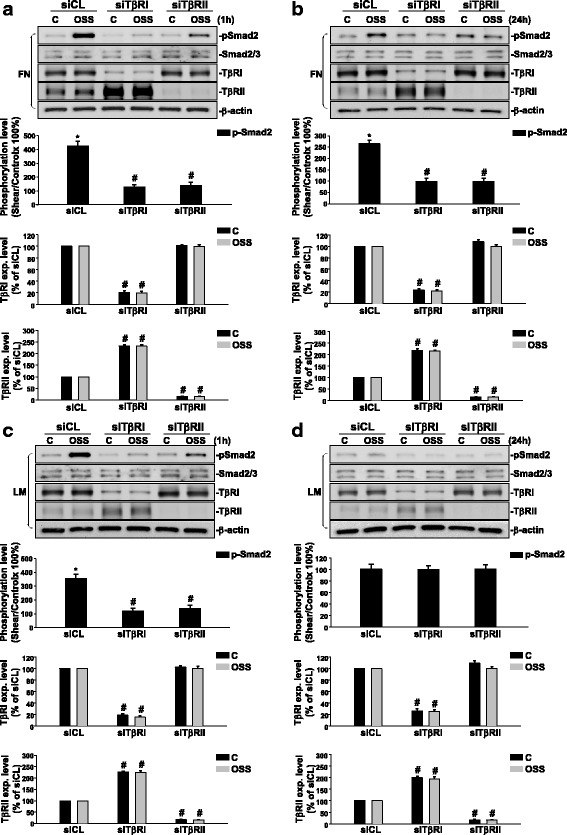
OSS-activation of Smad2 is regulated by TβRII and TβRI in ECs plated on FN and LM, respectively. ECs plated on FN (**a** and **b**) or LM (**c** and **d**) were transfected with control siRNA (siCL) or specific siRNA (30 nM each) of TβRI (siTβRI) and TβRII (siTβRII) for 48 h. The cells were kept under static conditions (C) or subjected to OSS (0.5 ± 4 dyn/cm^2^) for 1 h (**a** and **c**) or 24 h (**b** and **d**). The phosphorylation of Smad2 and expressions of TβRI and TβRII were determined by using Western blot analysis. Data are means ± SEM from three independent experiments. ^*^P < 0.05 vs. static control cells. ^#^*P* < 0.05 vs. siCL-transfected cells

To investigate which TGFβ receptor is responsible for OSS-induced Smad2 phosphorylation in ECs on different ECMs, ECs were transfected with TβRI- or TβRII-specific siRNAs (30 nM), and kept under static conditions or exposed to OSS for 1 h or 24 h. Transfection of ECs with TβRI- or TβRII-specific siRNAs, which caused approximately 80% and 90% reductions (compared with control siRNA) in TβRI and TβRII protein expressions, respectively, significantly inhibited OSS-induced Smad2 phosphorylation in ECs plated on both FN (Fig. 4a) and LM (Fig. 4c) after shearing for 1 h. Moreover, transfections of ECs with TβRI- or TβRII-specific siRNAs (compared with control siRNA) showed significant inhibitions in OSS-induced Smad2 phosphorylation in ECs plated on FN after shearing for 24 h (Fig. 4b), with no effects on Smad2 signaling in ECs plated on LM (Fig. 4d). These results indicate that the OSS-induced Smad2 phosphorylation in ECs plated on either FN or LM are both regulated by TβRI and TβRII.

### Oscillatory flow induces sustained interaction between integrins and TGFβ receptors in ECs plated on FN, but only transient interaction between integrins and TGFβ receptors in ECs plated on LM.

**Fig. 5:**
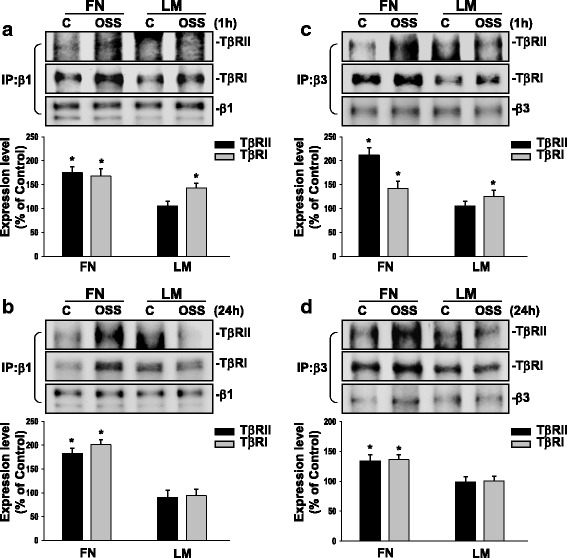
OSS induces sustained associations of TβRs and integrins in ECs plated on FN, but only transient associations of these molecules in ECs plated on LM. ECs plated on FN or LM were kept under static conditions (C) or subjected to OSS (0.5 ± 4 dyn/cm^2^) for 1 h (**a** and **c**) or 24 h (**b** and **d**). The cells were lysed to determine the interaction of β1 (**a** and **b**) and β3 (**c** and **d**) integrins with TβRII and TβRI by using immunoprecipitation assay. Data are means ± SEM from three independent experiments. ^*^P < 0.05 vs. static control cells

To further investigate whether TGFβ receptors can interact with integrins to activate EC Smad2 on different ECM proteins in response to OSS, we performed co-immunoprecipitation assays with an antibody against β1 or β3 integrin, followed by Western blot analysis with antibodies against TβRI and TβRII. Our co-immunoprecipitation results showed that application of OSS for 1 h to ECs plated on either FN or LM could enhance the interactions between β1 (Fig. 5a) and β3 integrins (Fig. 5c) with TβRI and TβRII, as compared with static controls. These OSS-induced associations were sustained for 24 h only in ECs plated on FN, but not on LM (Fig. 5b and Fig. 5d). These findings indicate that OSS-activation of Smad2 in ECs plated on both FN and LM is regulated by interactions between β1 and β3 integrins and TβRs, and the time course of OSS-activation of Smad2 depends on the interactions between integrins and TβRs.

### Smad2 regulates OSS-induced NF-κB and pro-inflammatory gene expression in ECs plated on FN or LM.

**Fig. 6:**
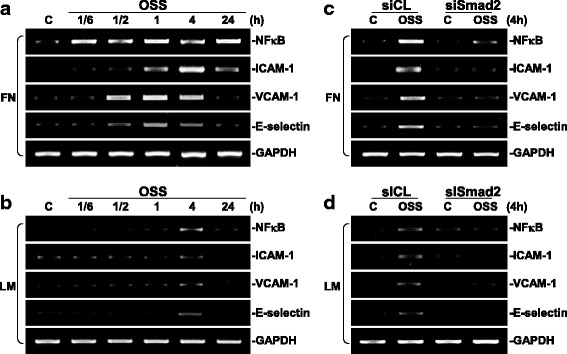
Smad2 regulates OSS-induced NF-κB signaling in ECs. ECs plated on FN (**a**) or LM (**b**) were kept under static conditions (C) or subjected to OSS (0.5 ± 4 dyn/cm^2^) for 1/6 h (10 min), 1/2 h (30 min), 1 h, 4 h and 24 h, and their mRNAs were collected to detect the expressions of NF-κB, ICAM-1, VCAM-1, and E-selectin by using RT-PCR analysis. ECs plated on FN (**c**) or LM (**d**) were transfected with control siRNA (siCL) or specific siRNA (30 nM) of Smad2 for 48 h. The cells were kept under static conditions (C) or subjected to OSS (0.5 ± 4 dyn/cm^2^) for 4 h. The expressions of NF-κB, ICAM-1, VCAM-1, and E-selectin were examined by using RT-PCR analysis. These results are representative of three independent experiments with similar results

OSS has been shown to play important roles in the modulation of EC inflammation [1, 8]. We assessed whether OSS could regulate the expression of NF-κB and pro-inflammatory adhesion molecules, including ICAM-1, VCAM-1, and E-selectin, in ECs plated on different ECM proteins. The results showed that OSS induces the expression of pro-inflammatory genes, including NF-κB, ICAM-1, VCAM-1, and E-selectin, in ECs plated on FN. Among these genes, NF-κB and ICAM-1 expressions were sustained over the 24 h period tested, whereas VCAM-1 and E-selectin expressions returned to the basal level within 24 h (Fig. 6a). On the other hand, OSS could only slightly induce the expression of these pro-inflammatory molecules in ECs plated on LM (Fig. 6b). These OSS-inductions of pro-inflammatory genes reached the maximal levels within 4 h and returned to the basal levels after 24 h of shearing. Transfections of ECs with Smad2-specific siRNAs (30 nM, compared with control siRNA) abolished these OSS-induced pro-inflammatory gene expressions in ECs plated on either FN (Fig. 6c) or LM (Fig. 6d), indicating that Smad2 is critical for OSS-induced pro-inflammatory responses in ECs grown on different ECM proteins.

### Smad2 is highly activated in ECs of human atherosclerotic coronary arteries, which is accompanied by the concomitant increase in FN expression in ECs and neointimal region.

**Fig. 7:**
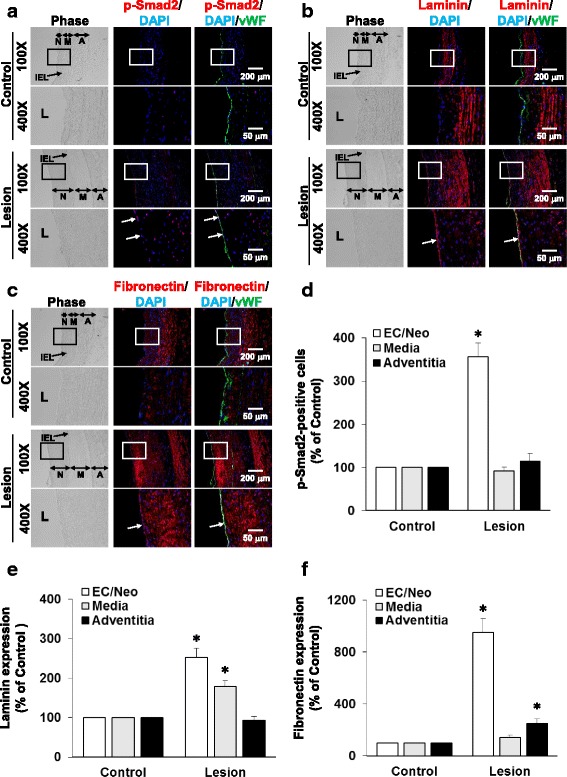
Phospho-Smad2 is pronouncedly stained in the EC layer of human coronary atherosclerotic lesions. Serial cross-sections of diseased human coronary arteries and control internal thoracic arteries from same patients were stained for human phospho-Smad2 (**a**), laminin (**b**), and fibronectin (**c**) and counterstained with vWF and DAPI. Arrow, phospho-Smad2-positive cells or the EC layer; IEL: internal elastic lamina; L: lumen; N: neointima; M: media; A: adventitia. Pictures in 400X are the magnified views of the indicated areas (white line box) in the 100X pictures. (**d**) Quantitative analysis of the results in **a** showing the percentage of numbers of phospho-Smad2-positive cells relative to DAPI-stained cells. Data are means ± SEM of ~150 cells for EC layer and ~200 cells for each other regions from three to five independent experiments. (**e** and **f**) Quantitative analysis of the results in **b** and **c**, respectively. Images shown in each examination are representative of five experiments with similar results. Data are means ± SEM from five independent experiments. ^*^P < 0.05 vs. control vessels

To access the activation of Smad2 in atherosclerotic lesions, immunohistochemical examinations were made on the diseased human coronary arteries, with the use of internal thoracic arteries from the same patients as controls. There is a pronounced staining of phospho-Smad2 in the cell nuclei in the EC layer (indicated by arrows), but not the neointimal, medial, and adventitial regions in diseased arteries (Fig. 7a and d). In contrast, the control thoracic aorta shows virtually no detectable staining of phospho-Smad2 in the EC layer. Immunohistochemical staining of serial sections showed that laminin is highly expressed in the medial layers of both control and atherosclerotic arteries, and is also positively stained in the EC layer of atherosclerotic lesions (Fig. 7b and e). In contrast, fibronectin is mainly expressed in the adventitial region of both control and atherosclerotic arteries, and its expression is significantly increased in the EC layer and neointimal region in atherosclerotic lesions (Fig. 7c and f). These results indicate that Smad2 is highly activated in ECs of human atherosclerotic coronary arteries, which is accompanied by the concomitant increase in the expression of FN, rather than LM, in the EC layer and neointimal region in atherosclerotic lesions.

## Discussion

**Fig. 8:**
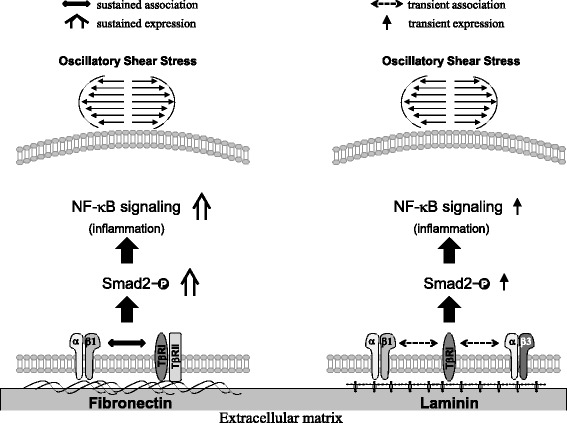
Schematic representation of the signaling pathways. OSS induces a sustained increase in the association of TβRII and TβRI with integrin β1 and β3 in ECs plated on FN, but only induces a transient association in ECs plated on LM. These differential effects of FN and LM on EC responses to OSS may contribute to the differential regulations of these ECM proteins in Smad2 and NF-κB activations and downstream pro-inflammatory gene expression in ECs in response to OSS

The aim of this study is to investigate the mechanism by which different ECM proteins, i.e., FN and LM, play differential roles in the modulation of Smad2 activation and hence pro-inflammatory signaling in ECs in response to disturbed flow. These differential regulations of FN and LM in EC pro-inflammatory responses to disturbed flow may be involved in the formation and progression of atherosclerosis (summarized in Fig. 8). Several lines of evidence support these findings. First, OSS induces a sustained activation of Smad2 in ECs grown on FN, but it only induces a transient activation of Smad2 in ECs on LM. Second, β1 and β3 integrins can be activated by OSS in ECs on FN, but only β1 integrin is involved in OSS-activation of Smad2 in these ECs. In contrast, both β1 and β3 integrins are involved in OSS-induced transient activation of Smad2 in ECs on LM. Third, both TβRI and TβRII are involved in OSS-activation of Smad2 in ECs grown on either FN or LM, since knockdowns of these TGF-β receptors are able to abolish OSS-activation of Smads in these ECs. Fourth, the OSS-induced sustained activation of Smad2 in ECs on FN may be attributable to the sustained association of β1 integrin with TβRI and TβRII. In contrast, OSS can only induce a transient association of β1 and β3 integrins with TβRI in ECs grown on LM. Fifth, OSS induces sustained expressions of NF-κB and ICAM-1 in ECs on FN, but it only can induce a transient expression of all examined pro-inflammatory genes in ECs on LM. These responses can be regulated by Smad2. Finally, examinations of diseased human coronary arteries from CAD patients under revealed that Smad2 is highly activated in ECs of human atherosclerotic coronary arteries, which is accompanied by the concomitant increases in the expression of FN, rather than LM, in the EC layer and neointima in lesions. These findings indicate that EC Smad 2 is a critical mechano-transducer from the association of integrins and TGF-β receptors, whose activation is differentially regulated by different ECM proteins, i.e., FN and LM, and may consequently contribute to the formation and progression of atherosclerosis in the disturbed flow region of arterial trees.

ECM compositions have been implicated to be associated with the development of atherosclerosis. Previous in vivo studies have shown that FN deposited into sub-endothelial space at the atherosclerosis-prone regions of aortic arches correlates with the activation of pro-inflammatory signaling pathways attributable to regional hemodynamic conditions [17, 18]. Recent in vitro studies have also shown that disturbed flow induces platelet-endothelial cell adhesion molecule-1 expression, which further enhances FN deposition and NF-κB activation in ECs [18]. These findings provide an evidence that ECM proteins play important roles in the modulation of pro-inflammatory signaling pathways in ECs in response to disturbed flow and contribute to the formation and progression of atherosclerosis. In the present study, we used LM and FN as model ECM proteins to mimic the situations in the normal vascular basement membranes and atherosclerotic subendothelial matrices, respectively. We focused on clarifying how LM (physiological condition) and FN (pathophysiological condition) affect the activation of Smad2 and their downstream pro-inflammatory signaling in ECs in response to OSS. Smad2 is known as a signaling molecule that can modulate a variety of signaling networks and cellular functions [13]. It has been shown to contain three domains: the conserve N-terminal MH1 domain, the linker domain, and the C-terminal MH2 domain [19, 20]. A recent study indicates that laminar shear stress can activate Smad2 in the linker region but does not induce Smad2 phosphorylation in the C-terminal region [13]. However, the C-terminal MH2 domain is essential for the dimerization of Smad protein and its nuclear transport, transcriptional activation, and interaction with type I receptors [21, 22]. In the present study, we demonstrated that disturbed flow can induce a transient phosphorylation of Smad2 in the C-terminal MH2 domain when ECs are adhered to the major ECM component of basement membrane (i.e., LM). However, OSS induces a sustained increase of Smad2 phosphorylation in the C-terminal MH2 domain in ECs plated on the major ECM deposited in atherosclerotic lesions (i.e., FN). Our results indicate that Smad2 can be differentially activated in the MH2 domain in ECs under disturbed flow on different ECMs that are corresponding to the physiological or pathophysiological conditions of normal and atherosclerotic vascular walls. These findings suggest that the sustained activation of Smad2 in ECs surrounded by FN may be an important indicator for the development of atherosclerosis.

Integrins are α/β heterodimeric proteins in which a β integrin may associate with different α form of integrins to manifest various functions. Shear stress-induced mechanotransduction in ECs is known to require integrin activation [6]. β1 and β3 integrins have been shown to be the principal receptors for ECs anchored to ECMs, including FN and LM [23, 24]. Our previous studies demonstrated that atheroprone flow with OSS can induce sustained activation of β3 integrin in ECs plated on FN, whereas LSS at 12 dynes/cm^2^ to mimic the physiological levels of flow can only induce a transient activation of β3 integrin in ECs on FN [15, 16]. In addition, these shear-induced sustained and transient activations of β3 integrin exerted differential effects on Smad1/5 activation in ECs [15, 16]. In this study, we further demonstrated that OSS induces sustained activations of β1 and β3 integrins in ECs plated on FN, whereas it can only induce a transient activation of these integrins in ECs plated on LM. These results suggest that OSS-induced differential activation of Smad2 in ECs on FN vs. LM could be attributable to the differential regulation of these ECM proteins in integrins activation. The mechanisms of crosstalk between different integrins in the regulation of cell signaling have been established [25, 26]. Recent studies demonstrated that functions of β3 integrin are regulated by β1 integrin on ECs and their crosstalk is shown to modulate angiogenesis [26, 27]. Under normal conditions, β1 and β3 integrins were shown to cooperate to modulate angiogenesis of ECs adhered to LM [24]. In concert with this previous study, our current data also showed that OSS-induced transient activation of Smad2 in ECs on LM is modulated by both β1 and β3 integrins, whereas OSS-induced sustained activation of Smad2 in ECs on FN is only regulated by β1, but not β3, integrin. Our data advanced the notion that differential crosstalk between β1 and β3 integrins for different ECM proteins may be critical for EC responses to physiological and pathophysiological conditions. Our findings also implicate that β1 and β3 integrins may play different roles in the modulation of mechanotransduction in ECs grown on different ECMs in response to pathophysiological stimuli.

A recent study showed that collagen-induced Smad 2/3 and Src activations are mediated by soluble ECM peptides, and these activations require the interaction between collagen receptor and TGF-β type I and type II receptors [28]. These previous findings led us to assess the association between OSS-mediated integrins and TβRs. Our results showed that exposure of ECs plated on FN to OSS for 24 h could induce sustained activations of β1 and β3 integrins, which were able to associate with TβRI and TβRII. However, exposure of ECs plated on LM to OSS could only induce transient associations of β1 and β3 integrins with TβRI, but not TβRII. Furthermore, transfections of ECs with TβRI- and TβRII-specific siRNAs eliminated the OSS-activation of Smad2 in ECs plated on either FN or LM. These findings support the notion that TβRs may serve as mechanoreceptors to cooperate with integrins to transduce mechanical stimuli into intracellular Smad signaling in ECs in response to disturbed flow.

Atherosclerosis is a chronic inflammatory vascular pathology leading to various vascular diseases. The activation of NF-κB transcription factor plays important roles in regulating the expression of pro-inflammatory molecules, including ICAM-1, VCAM-1, and E-selectin [18, 29]. Smads have recently emerged as important signaling molecules to cooperate with NF-κB to modulate pro-inflammatory gene expression [30]. Thus, we investigated whether Smad2 could regulate NF-κB signaling and these pro-inflammatory genes in ECs plated on FN or LM in response to OSS. Recent studies by Orr et al. [29] showed that laminar shear stress can only slightly up-regulate NF-κB in ECs plated on LM after shearing for 1/2 h. Sorescu et al. [31] demonstrated that OSS regulates the EC expression of ICAM-1, rather than VCAM-1 and E-selectin. Chappell et al. [32] showed that OSS up-regulates ICAM-1, but not VCAM-1, mRNA after 24 h of shearing. Moreover, a recent in vivo study reported that human carotid artery bifurcation highly expresses immunoactivity of ICAM-1, but not of VCAM-1 or E-selectin [33]. In concert with these previous findings, our present study showed that among these pro-inflammatory genes, the expressions of NF-κB and ICAM-1 in ECs on FN could be induced by OSS in a sustained manner (i.e., 24 h). In contrast, OSS-induction of these four pro-inflammatory genes in ECs on LM declined and returned to the basal levels after 4 h of shearing. These results indicate that different ECM proteins not only can play differential roles in the modulation of EC Smad2 activation, but also exert differential effects on the mechanosensitivity of pro-inflammatory genes in ECs in response to disturbed flow.

One of the most interesting findings of our study is that up-regulation of Smad2 phosphorylation has strongly positive correlation with the induction of FN expression in the EC layer and neointimal region of atherosclerotic lesions in the diseased human coronary arteries. As expected, our immunostaining results on normal arteries showed that LM is highly expressed in the medial layer, but not the neointimal and adventitial regions, in the arteries. In contrast, FN was mainly expressed in the adventitial, but not neointimal and medial, regions. There was virtually no detectable staining of phospho-Smad2 in ECs. However, significant increase in phospho-Smad2 was found in the EC layer of atherosclerotic lesions, which was accompanied by the dramatically concomitant increase in the expression of FN, but not LM, in the EC layer and neointimal region in lesions. These results indicate that FN signaling surrounding ECs may play important roles in the activation of Smad2 signaling in lesion ECs, which may consequently contribute to the formation and progression of atherosclerosis.

## Conclusions

Our present study has provided new insights into the mechanisms by which different ECM proteins, i.e., FN and LM, exert differential effects on Smad2 activation and hence pro-inflammatory gene induction in ECs in response to disturbed flow. OSS induces sustained phosphorylation of Smad2 through sustained activation of β1 integrin and its association with TβRI and TβRII in ECs plated on FN, but it only induces transient phosphorylation of Smad2 in ECs plated on LM, which may be attributable to the transient activations of β1 and β3 integrins and their association with TβRI. These differential effects of FN and LM on EC Smad2 activation may contribute to their differential regulation in pro-inflammatory gene expression in ECs in response to OSS. The importance of FN-associated EC Smad2 activation in atherogenesis is shown by our immunohistochemical examinations on the diseased human coronary arteries. Our findings have characterized TβR-specific Smad2 as a connecting link for the chain of events of disturbed flow, mechanical sensing, EC inflammation, and atherogenic responses in the arterial wall. Such findings may provide insights to therapeutic intervention against disturbed flow-associated vascular pathologies, e.g., atherosclerosis.
